# Trichosporon asahii fungemia and COVID-19 co-infection: An emerging fungal pathogen; case report and review of the literature

**DOI:** 10.1016/j.idcr.2021.e01244

**Published:** 2021-08-03

**Authors:** Gawahir A. Ali, Ahmed Husain, Husam Salah, Wael Goravey

**Affiliations:** a Department of Infectious Diseases, Communicable Diseases Centre, Hamad Medical Corporation, Doha, Qatar; b Department of Laboratory Medicine and Pathology, HMC, Doha, Qatar

**Keywords:** COVID-19, Trichosporon asahii, Fungemia, Voriconazole, Central venous catheters

## Abstract

With the evolving COVID-19 pandemic, increasing concerns about invasive fungal infections have been reported particularly with the use of potent immunosuppressant medications to treat the immunological storms in patients with severe COVID-19 illnesses. Trichosporon asahii (T. asahii) is an emerging highly resistant pathogen with considerable mortality particularly in critically ill patients and immunocompromised individuals. We describe a case of a 58-year-old patient who developed T. asahii fungemia after using immunosuppressant agents for his severe COVID-19 related cytokines release syndrome. Pseudohyphae, arthroconidia, and lateral blastoconidia were seen in the stain, and later confirmed to be T. asahii. Voriconazole successfully treated this multi-drug-resistant fungal infection. The clinical presentation, assessment, and management are reviewed to raise awareness of the circumstances leading to coinfection with this emerging resistant yeast.

## Highlights

• Trichosporon asahii fungemia is an emerging invasive pathogen in critically ill Coronavirus disease 2019 patients.
• Judicious use of immunosuppressant medications is advisable in critically ill Coronavirus disease 2019.
• Periodically fungal surveillance program is recommended in critically ill COVID-19 patients.
• Voriconazole is the first option of therapy for Trichosporon asahii infection.

## Introduction

Trichosporon is a non-Candida, environmental yeast-like basidiomycete, an emerging fatal opportunistic fungal infection that predominantly occurs in immunocompromised individuals, particularly in hematological malignancies [1]. Trichosporon asahii is the most common fungus in this genus and causes considerable mortality [2]. Other risk factors for invasive trichosporonosis are antibiotic use, AIDS, use of corticosteroids, long ICU stay, presence of central venous catheters (CVC), and invasive medical equipment [1]. The emergence of the COVID-19 pandemic and the development of catastrophic immunological storms in patients with severe COVID-19 illnesses have promoted the use of various therapeutic interventions including immunosuppressive therapy [3], [4]. Thus, provoking the emergence of latent and opportunistic coinfections including multidrug-resistant organisms and fungal infections [5]. Timely identification of Trichosporon fungemia and early Voriconazole use is crucial to avoid ominous outcomes [1].

To highlight this, we describe a case of Trichosporon asahii fungemia in a critically ill COVID-19 patient discussing relevant associated risks leading to the event and aiming to raise awareness regarding the circumstances and the emergence of this multi-drug resistant opportunistic pathogen during the COVID-19 pandemic and review the literature for similar cases.

## Case description

**Fig. 1:**
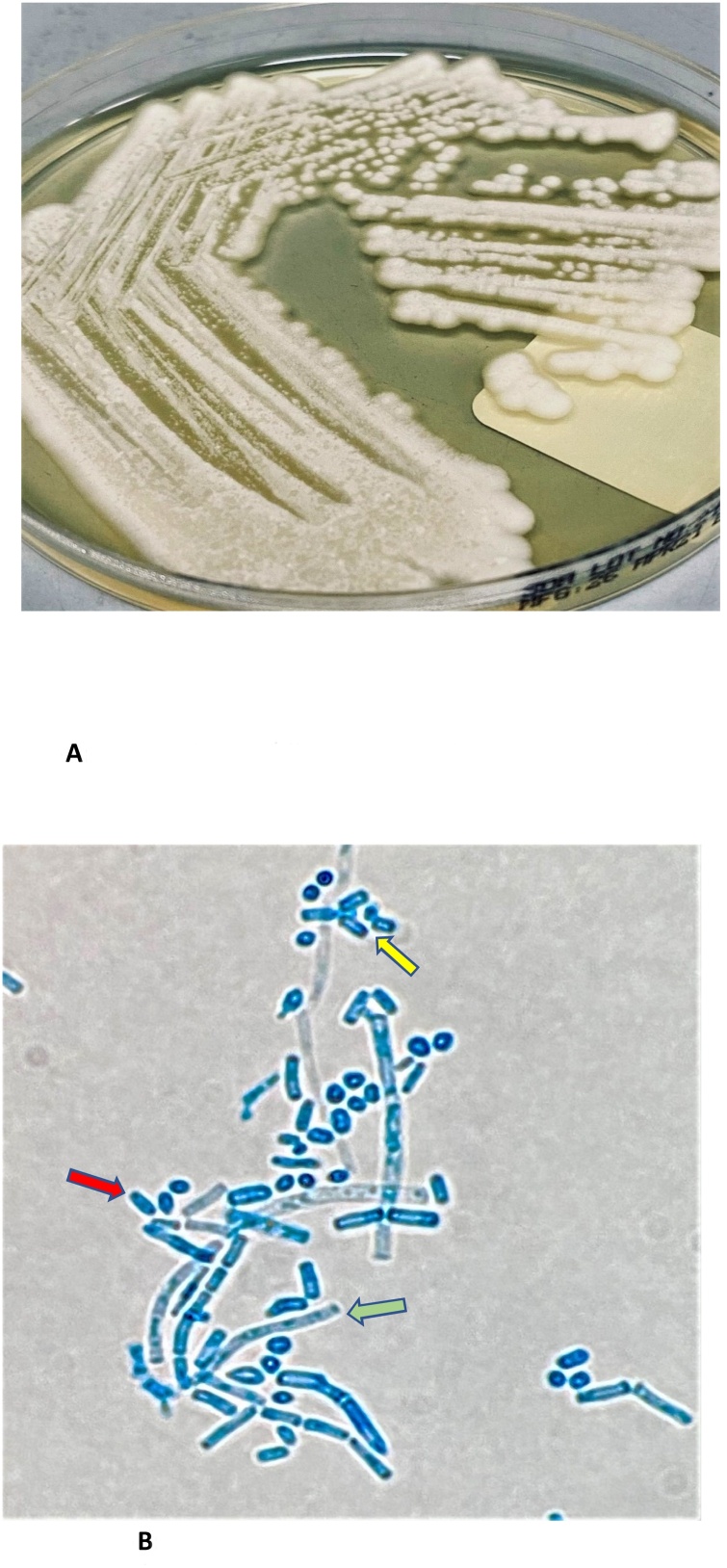
**A**: Colony of *T. asahii* on Sabouraud’s dextrose agar after 48 h incubation at 37 °C. **B**: Microscopic features of *T. asahii* with pseudohyphae (green arrow), arthroconidia (yellow arrow) and lateral blastoconidia (red arrow), (Lactophenol Cotton Blue stain, ×100).

A 58-Year-old man with well-controlled diabetes and hypertension presented to our emergency department with symptoms of fever, cough, and progressive shortness of breath for 3 days. Chest x-ray revealed bilateral infiltrates, and the diagnosis was subsequently confirmed as COVID-19 pneumonia by a positive SARS-Cov2 PCR. Shortly after admission, the patient deteriorated, and laboratory tests showed white blood cells of 3.6 × 10^9^/L (4–11.0) with profound lymphopenia at 0.2 × 10^9^/L (1–3), thrombocytopenia 30 × 10^9^/L (150–400), elevated inflammatory markers, including CRP 120 (0−5 mg/L), IL6 120 pg/mL (normal < 8) and ferritin 990 u/L (30−553). His renal functions were within the normal limit, but d-dimer was 3.25 mg/L (0−0.44) and APTT prolonged to 54.3 s (9.7–11.8). He progressed to ARDS, clinically assessed as COVID-19 related Cytokine Storm (CCS). The patient was admitted to the intensive care unit (ICU) and the oxygenation was maintained with continuous positive airway pressure therapy (CPAP) and eventually intubated. He was treated with steroids as well as an IL-6 inhibitor, Tocilizumab (total dose of 1200 mg, in two separate doses) in addition to broad-spectrum antibiotics, piperacillin-tazobactam, and Favipiravir as per local hospital policy with a remarkable dropping in the inflammatory markers. However, on day 11 of hospital admission, the patient developed fever with obvious sources of infection. Laboratory findings revealed a white cell count of 21.4 × 109/L (4–11.0) with 88 % neutrophils and a C-reactive level of 305 (0−5 5). Shortly after, yeast-like fungus was identified in the blood. Lactophenol cotton blue stain demonstrated pseudohyphae, arthroconidia, and lateral blastoconidia (Fig. 1 A and B). Subsequently, the mass spectrometry assisted by flight time desorption/ionization matrix (MALDI TOF-MS, Billerica, MA, USA) identified the yeast as T. asahii. CVC was removed and intravenous Voriconazole 6 mg/kg/ twice daily for two doses, then 4 mg/kg twice daily for two weeks with rapid clinical response and negative repeated culture. Isolated Trichosporon asahii proved to be sensitive to Voriconazole only and resistant to all other agents. The patient was extubated successfully on day 24 and discharged uneventfully from the hospital.

## Discussion

Trichosporon spp are yeast-like fungi that are present in the natural flora of the human body; however, they can become pathogenic with a wide spectrum of infections, ranging from superficial white piedra in immunocompetent individuals to fungemia and other invasive trichosporonosis in the immunocompromised host [2]. T. asahii is the most commonly identified pathogen in the genus with more than 70 % reported mortality in invasive trichosporonosis [1]. The 30-day mortality of COVID-19 related T. asahii fungemia was 80 % despite Voriconazole therapy [6]. With the evolving COVID-19 pandemic, increasing concerns about invasive fungal infections have been reported particularly with increased evidence in using steroids and Tocilizumab [5], [7]. Hence, a periodically fungal surveillance approach is recommended in critically ill COVID-19 patients [7]. Of note, patients with critical COVID-19 disease pose many risk factors for acquiring T. asahii fungemia as in our patient [1], [3]. The fact that T. asahii fungemia was grown from the CVC and not from the peripheral line might support that the CVS was the port of entry. In addition, T. asahii has a high ability to colonize the skin and the environment, including medical devices, leading to significant mortality and devise loss [8].

The matrix-assisted laser desorption ionization (MALDI) TOF MS demonstrates the ability to identify clinical Trichosporon isolates at the species level [9]. The management of T. asahii fungemia is challenging and difficult to treat but reversing the predisposing factors if possible and selecting the Voriconazole as a primary drug of choice could remarkably decrease mortality [1].

Our literature search yielded 7 cases of infection due to T. asahii in COVID-19 [6], [10], [11]. Rodriguez et al. reported one case of T. asahii fungemia in 20 cases of fungemia in COVID-19 patients with an overall reported mortality of 60 % in that cohort [10]. Gonzalo et al. reported a second case of T. asahii pneumonia in a COVID-19 patient [11]. Of note, this patient also had coinfection with Pseudomonas sp and Stenotrophomonas sp. and received steroids in addition to Tocilizumab as part of COVID-19 management but eventually succumbed to death. Almeida J et al. reported the remaining 5 cases in three months cohort duration with an average time of 23 days intensive care unit hospitalization to develop T. asahii fungemia. Noteworthy, this cohort did not show evidence of horizontal transmission raising the concern for the susceptibility of critically ill COVID-19 patients to T. asahii fungemia [6]

## Conclusion

Trichosporon asahii fungemia is an emerging invasive pathogen in critically ill Coronavirus disease 2019 patients that require attention due to high mortality and unique resistance profile. The need to be vigilant and actively screening this immunosuppressed population for rare and common fungal infections is paramount. Judicious use of immunosuppressant medications is advisable in such types of patients. Reversal of risk factors with an early selection of Voriconazole as the first option of therapy are crucial steps to prevent potential consequences.

## Declaration of Competing Interest

The authors report no declarations of interest.

## Funding

No funding was received towards the publication.

## Consent

A written informed consent was obtained from the patient to include clinical presentation together with results and imaging. This was subsequently reviewed and approved by the institution ethics and research review board with MRC0421544.

## Author contributions

GA: contribute to data acquisition and manuscript preparation. AH: Clinical management, data acquisition and manuscript writing. HS: contributed to data acquisition and Microbiology reports. WG: Corresponding author, clinical management, contribute to data acquisition and manuscript preparation.

## Compliance with ethical standards

Ethics approval and permission was obtained to publish the case reports from the institutional review board which is in line with international standards, MRC0421544.

## Data availability statement

The authors confirm that the datasets supporting the findings of this case are available from the corresponding author upon request.
